# Heterotopic Heart Transplant History and Concepts Cannot Be Neglected - Witnessing the History and Learning with Previous Practices

**DOI:** 10.21470/1678-9741-2021-0013

**Published:** 2021

**Authors:** Luciana F da Silva, Jose P da Silva

**Affiliations:** 1 Heart and Vascular Institute, UPMC Children’s Hospital of Pittsburgh, Pittsburgh, Pennsylvania, United States of America.

Dear Editor,

We read with interest the article published by Gaiotto et al.^[1]^ about a proposal for heterotopic heart transplantation (HHT). It has called our attention that the authors are recommending to suture the pulmonary artery (PA) to the right atrium (RA), closing the donor inferior vena cava, as originally reported by Barnard and Losman^[2]^ in their initial clinical experience, in 1974, with HHT as assistance to left ventricular failure. Those authors^[2]^ predicted in their paper, published in 1975, that “This technique can be used as permanent assistance in patients with extensive irreversible damage of the left ventricular muscle. On the other hand, it can be used as a temporary device to assist the left ventricle in patients whose lives are threatened by a reversible condition, for example, cardiogenic shock after myocardial infarction.”. However, this technique has the disadvantage of not supporting the right ventricle (RV) circulation when the recipient’s heart fibrillates or the recipient RV function deteriorates, being not applicable to biventricular dysfunction.

**Fig. 1:**
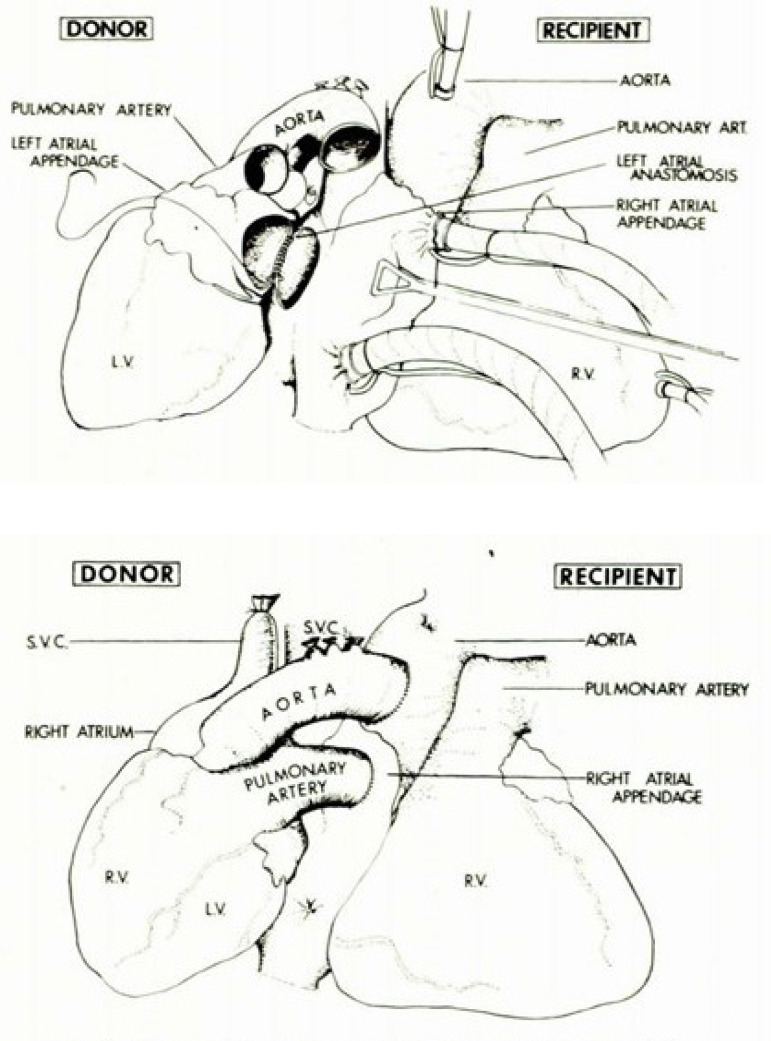
The original illustration of Barnard heterotopic heart transplant procedure for left ventricle support. Copyright permission fee to reproduce images obtained from South African Medical Association^[2]^

**Fig. 2:**
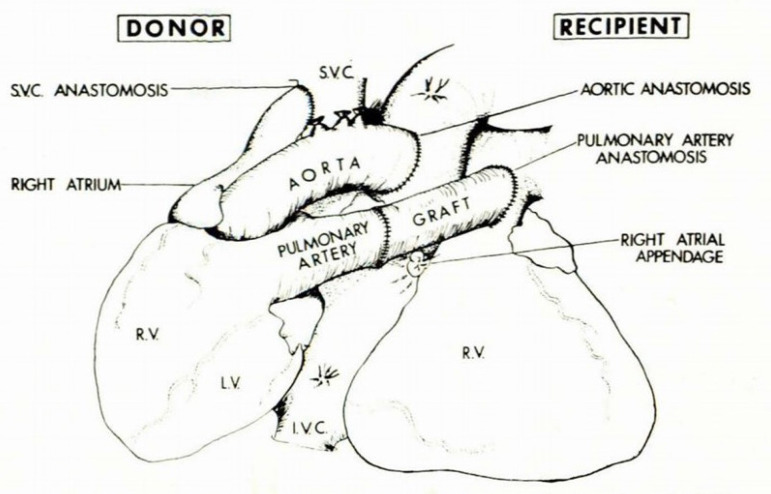
Barnard’s original illustration of biventricular support with the heterotopic heart transplant. Copyright permission fee to reproduce images obtained from South African Medical Association ^[2]^.

Barnard and Losman also wrote about assisting the RV by anastomosing the stump of the donor superior vena cava (SVC) to the recipient SVC in an end-to-lateral anastomosis, making the anastomosis of the PA donor to the PA recipient, creating a parallel heart circulation. Figures 1 and 2 reproduce their original article illustration with left ventricular and biventricular support, respectivelly, with the heterotopic heart. Therefore, the method described by Copeland^[3]^ is a mixing of two techniques applied in 1974, and it is not new, being previously employed and published by the Yacoub team, associated with myocardial revascularization^[4]^.

This method demonstrated shortcomings, so after two cases, Novitzky, Cooper, and Barnard adopted the biventricular support HHT technique completing systemic venous return connection by the side-to-side donor and recipient RA anastomosis, and the donor PA was connected to the recipient PA by the interposition of a Dacron conduit^[5]^.

To prevent the potential complications of a PA prosthetic conduit (infection, thrombosis, and obstruction with fibrosis tissue formation) and to avoid the heavy adhesions involving the recipient PA, in 1993, Da Silva et al. performed and published a new technique that allowed the connection of the donor PA to the recipient right PA without a conduit interposition and direct end-to-end SVC anastomosis. Endomyocardial biopsy is in this way facilitated because the SVC anastomosis easily leads the bioptome forceps to the transplanted RV^[6,7]^.

After that initial patient, we applied the same technique in three other situations. The long-term survival was 25 years in the first patient and seven and a half years in the second case; in both, the HHT indication was elevated pulmonary vascular resistance (PVR) in a set of cardiomyopathy^[7]^. In two subsequent patients, the HHT was indicated as assistance to biventricular function recovery after congenital heart disease repair. After six and a half years of her heart transplant, the third patient is alive and in good clinical condition. The fourth patient had explantation of the donor’s heart 11 months after HHT, with full recovery of his native heart function, growing in excellent clinical condition four and half years after his last surgical procedure. Therefore, the current proposal^[1]^is not new in this aspect either.

Copeland^[3]^ has used the combination of the two initial HHT approaches described by Barnard^[2]^, then keeping the SVC’s partial blood flow directed to the RA. This donor PA to recipient RA anastomosis prevents its use in patients with biventricular failure^[1,4]^. Therefore, Copeland et al.^[3]^ and Gaiotto et al.^[1]^ are suppressing the benefit of donor RV support in future recipient’s arrhythmic events or progressive right ventricular dysfunction, reducing the likelihood of long-term survival with HHT, which in many reports has been demonstrated to be possible^[7-12]^.

The clot formation mentioned by authors in HHT usually occurs in the recipient left ventricle (LV)^[9]^. The impaired ventricular contractility against a normal systemic pressure generated by the donor LV causes a low blood flow velocity inside the enlarged recipient LV with a predisposition to thrombogenesis. Clot formation will not be modified by a right-side anastomosis modification, except as we did, by excluding conduit graft interposition, directly anastomosing the donor PA to the recipient right PA.

The potential benefit of HHT functioning as a temporary ventricular assist device (or VAD) was predicted by Drs. Barnard and Losman in 1975 and demonstrated in one of our patients and by other author^[10]^. Our HHT approach has demonstrated excellent clinical outcomes and allowed biventricular function recovery.

Previously described HHT techniques do not preclude the idea of subsequently rotating the recipient heart to a central position. However, it will be extremely rare to confront a situation where the transplanted patient has a clinical indication to remove his native heart in clinical practice. The PVR needs to decrease enough to allow orthotopic heart transplant while the donated heart is still free of allograft coronary artery disease, justifying keeping the previously transplanted heart instead of replacing it with a new donor heart in orthotopic position. The argument that an RV connected to the RA will be adequate in such a situation is most unlikely since the RV managing SVC blood flow against a low RA pressure will be unprepared even to face normal PVR and can complicate the postoperative period.

Therefore, we conclude with a word of caution against this proposed technique.

The history of HHT has demonstrated valuable and safer ways to apply it and demands at least to be precisely alluded in modern era.
